# Adsorption of crystal violet and malachite green onto dibarium magnesium orthoborate (Ba_2_Mg(BO_3_)_2_): equilibrium, kinetics and thermodynamics

**DOI:** 10.1039/d6ra03351b

**Published:** 2026-10-06

**Authors:** Uğur Özkan, Okan Bayram, Fethiye Göde, İlhan Pekgözlü

**Affiliations:** a Department of Forest Products Engineering, Faculty of Forestry, Isparta University of Applied Sciences 32200 Isparta Turkey ugurozkan@isparta.edu.tr; b Department of Chemistry, Faculty of Engineering and Natural Sciences, Süleyman Demirel University 32200 Isparta Turkey okanbayram@sdu.edu.tr; c Department of Chemistry, Faculty of Engineering and Natural Sciences, Süleyman Demirel University 32200 Isparta Turkey fethiyegode@sdu.edu.tr; d Department of Environmental Engineering, Faculty of Engineering, Karabük University Karabük Turkey ilhanpekgozlu@karabuk.edu.tr

## Abstract

Dibarium magnesium orthoborate (Ba_2_Mg(BO_3_)_2_) is a borate based ceramic compound containing two different cations within its crystal structure. In this study, the synthesized Ba_2_Mg(BO_3_)_2_ was evaluated as an adsorbent for the removal of the cationic dyes malachite green (MG) and crystal violet (CRV) from aqueous solutions. The structural, morphological, surface and thermal properties of Ba_2_Mg(BO_3_)_2_ were characterized using X-ray diffraction (XRD), Fourier-transform infrared (FT-IR) spectroscopy, scanning electron microscopy coupled with energy dispersive X-ray spectroscopy (SEM-EDS), thermogravimetric analysis (TGA), Brunauer–Emmett–Teller (BET) surface area analysis, zeta-potential measurements and particle size analysis. Batch adsorption experiments were conducted to examine the effects of pH, initial dye concentration, adsorbent dosage, contact time and temperature on removal efficiency. The kinetic data were fitted to the pseudo-first-order (PFO), pseudo-second-order (PSO), and intra-particle diffusion models, with the PSO model providing the best fit for both dyes. Equilibrium data were evaluated using the nonlinear Langmuir, Langmuir Type II, Freundlich, Scatchard (Langmuir Type IV), and Temkin models. The nonlinear Langmuir model predicted *q*_max_ values of 2283.835 and 2327.393 mg g^−1^ for MG and CRV, respectively. Under the investigated conditions, Ba_2_Mg(BO_3_)_2_ showed high removal performance for both MG and CRV. However, adsorbent induced aggregation can also contribute to dye removal.

## Abstract 2

Dibarium magnesium orthoborate (Ba_2_Mg(BO_3_)_2_) is a borate based ceramic compound containing two different cations within its crystal structure.

## Graphical abstract

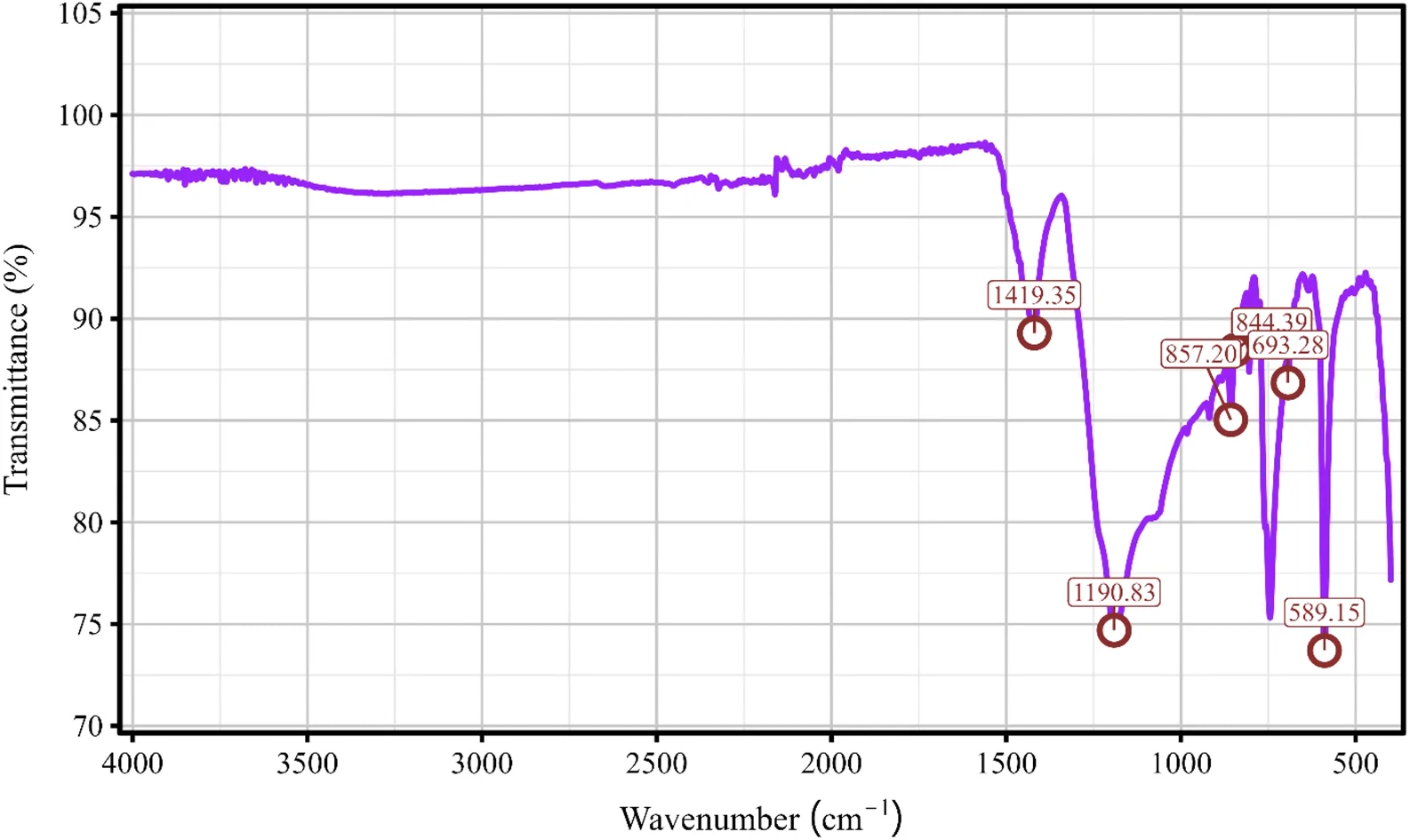

## Introduction

Dye containing industrial effluents are widely regarded as a major source of water pollution. Owing to their strong coloration and chemical stability, dyes markedly impair the aesthetic quality and usability of water resources. The continued expansion of industrial activities has led to increasing wastewater discharges from sectors such as textiles, paper, cosmetics, food processing, and leather manufacturing into receiving environments. Industrial effluents introduce a range of contaminants into aquatic systems, including heavy metal ions, fertilizer derived components, pharmaceutical residues, phenolic compounds, plastic waste and dyes all of which pose significant risks to living organisms.^1–9^ It has been reported that a significant fraction of dyes used during textile production enters wastewater streams, leading to contamination of water bodies.^10–12^ The general resistance of dyes to biodegradation further contributes to their persistence, exerting stress on microbial communities and disrupting ecological processes.^10–12^ Depending on their chemical structure and binding mechanisms, dyes are classified into various categories such as anionic, cationic, acidic, reactive, direct, basic, and azo dyes. Azo dyes account for more than 60% of commercially used dyes and are considered the most widely applied dye class in the textile industry.^12^ Owing to the stability of their azo linkages and aromatic structures, many azo dyes are poorly biodegradable, particularly under aerobic conditions, and may persist in receiving waters.^13^ Moreover, their degradation products have been reported to exert mutagenic effects on aquatic microorganisms and to disrupt nitrogen cycling processes.^14^ Crystal violet (CRV) is a commercially important cationic dye widely used in textile dyeing and various industrial applications.^15–17^ However, its presence in wastewater is of environmental concern, as exposure may cause adverse health effects ranging from skin and eye irritation to respiratory and renal disorders.^18,19^ The aromatic structure of CRV confers considerable chemical stability, enabling it to persist in aquatic and soil environments for extended periods.^20^ Similarly, Malachite Green (MG) is a cationic dye extensively employed in textile, paper, food, medical, aquaculture, and leather industries. Despite its widespread use due to low cost and stability in aqueous media, the discharge of MG containing effluents poses significant risks to human health and ecosystems because of its toxic and carcinogenic properties.^21–24^ Dye-contaminated wastewater reduces light penetration in aquatic environments, adversely affects gas solubility, and causes visual pollution, thereby negatively impacting aquatic life.^25–27^ To mitigate the environmental impact of such pollutants, various treatment technologies have been developed, including membrane filtration, coagulation–flocculation, electrodialysis, photocatalytic degradation, biological treatment, and adsorption.^28–33^ However, many of these methods are associated with limitations such as high operational costs, sludge generation, long processing times, or strict operating conditions.^34–37^ Adsorption is widely regarded as an attractive alternative owing to its operational simplicity, cost-effectiveness, and high removal efficiency, even at low pollutant concentrations.^38^ In recent years, inorganic metal borates have attracted attention as potential adsorbent materials because of their distinctive structural features and possible porous morphologies.^39,40^ Boron commonly occurs in trigonal planar BO_3_ units with approximate sp^2^ character and tetrahedral BO_4_ units with approximate sp^3^ character. These structural units significantly influence the physical and chemical properties of borate compounds.^41,42^ Among alkaline earth metal borates, dibarium magnesium orthoborate (Ba_2_Mg(BO_3_)_2_) has a crystal structure composed of BaO_9_ polyhedra, MgO_6_ octahedra, and BO_3_ triangular units.^43^ The compound has been extensively investigated for optoelectronic applications, and the optical properties of its doped forms are well documented.^44–47^ Some metal borates are also thought to exhibit porous structures that could be advantageous for adsorption applications. The present study investigates the use of Ba_2_Mg(BO_3_)_2_ as an adsorbent for removing the cationic dyes MG and CRV from aqueous solutions. The adsorption performance of the two dyes was comparatively evaluated. The structural and thermal properties of the material were comprehensively characterized by FT-IR, SEM-EDS, BET, XRD, TGA, zeta potential, and particle size analyses. The study aims to contribute to the existing literature by highlighting the potential of metal borate-based adsorbents for dye removal applications.

## Materials and methods

### Materials and reagents

The cationic dyes malachite green (MG) and crystal violet (CRV) were obtained from ISOLAB and Sigma-Aldrich, respectively. The concentrations of the dyes in aqueous solutions were determined using the UV-vis spectrophotometric method. The maximum absorption wavelengths were identified as 617 nm for MG and 592 nm for CRV, based on both experimental measurements and the manufacturer's specifications. Drying of the synthesized adsorbent was carried out using a Nüve FN500 oven to remove free moisture from the samples. Dye concentrations before and after the adsorption experiments were measured using DLAB SP-UV1100 UV-vis spectrophotometer. The pH values of the dye solutions were determined using a Hanna HI2020-02 edge pH meter. Fourier Transform Infrared (FT-IR) spectroscopy of Ba_2_Mg(BO_3_)_2_ was performed using a JASCO FT/IR-4700 Type A instrument to identify functional groups present in the material. The surface morphology, particle structure, and elemental composition were examined using a Quanta FEG-250 scanning electron microscope equipped with an energy-dispersive X-ray spectroscopy (SEM-EDS) system. Thermal stability and mass changes as a function of temperature were evaluated by thermogravimetric (TG) and differential thermogravimetric (DTG) analyses in the temperature range of 25–1400 °C under an inert nitrogen atmosphere at a constant heating rate, using a Hitachi STA 300 (Hitachi, Japan) instrument. Prior to analysis, the sample was degassed under vacuum (1 × 10^−2^ mmHg) at 90 °C for 60 min and subsequently at 350 °C for 240 min. N_2_ adsorption desorption measurements were performed at 77 K using a Micromeritics Gemini VII 5.03 analyzer. Particle size distribution was analyzed with a BT-9300Z laser particle size analyzer. Zeta potential measurements were conducted using a HORIBA SZ-100 instrument to determine the surface charge characteristics of the material under the applied measurement conditions. All mass measurements throughout the experimental procedure were performed using a Radwag AS220 analytical balance.

### Synthesis of Ba_2_Mg(BO_3_)_2_

Ba_2_Mg(BO_3_)_2_ was synthesized using Ba(NO_3_)_2_, Mg(NO_3_)_2_·6H_2_O, and H_3_BO_3_ as the metal and boron precursors. Urea (CO(NH_2_)_2_) was employed as a fuel to facilitate the combustion process during the reaction stage. The precursor amounts used for the synthesis of Ba_2_Mg(BO_3_)_2_ were determined according to the stoichiometric Ba : Mg : B ratio of 2 : 1 : 2. Ba(NO_3_)_2_, Mg(NO_3_)_2_·6H_2_O, H_3_BO_3_, and urea were used in amounts of 2.613, 1.282, 0.618, and 1.800 g, respectively. The starting chemicals were first weighed in stoichiometric ratios using an analytical balance. They were then dissolved in deionized water under continuous stirring until a clear and homogeneous solution was obtained. The solution was transferred to a porcelain crucible and gradually heated on a hot plate at approximately 100 °C to evaporate excess water and form a viscous precursor gel. The material was then subjected to pre-combustion at 500 °C for 15 min in a muffle furnace. Gas evolution occurred during this stage, resulting in the formation of a porous intermediate powder. After cooling to room temperature, the powder was gently ground to ensure homogeneity and placed in an alumina boat. It was further calcined at 900 °C for 6 hours to complete crystallization and eliminate residual organic species. After cooling, a white powder was obtained, and phase formation was evaluated by XRD. The synthesized adsorbent was stored in a desiccator and used for subsequent characterization and adsorption experiments without any additional modification.

### Batch adsorption experiments

The adsorption performance of the synthesized Ba_2_Mg(BO_3_)_2_ toward cationic dyes was evaluated using the batch adsorption method. The effects of key operational parameters, including pH, adsorbent dosage, contact time, temperature, and initial dye concentration, were systematically investigated. To examine the effect of pH, experiments were conducted at a constant temperature of 25 °C with a contact time of 120 min, using an initial dye concentration of 400 mg L^−1^ and an adsorbent dose of 0.01 g. The pH of the dye solutions was adjusted (0.1 M HCl, 0.1 M NaOH) within the range of 4.5–10.5. The influence of adsorbent dosage was studied at 25 °C and 120 min of contact time, with an initial dye concentration of 400 mg L^−1^. Adsorbent amounts of 0.005, 0.01, 0.015, and 0.02 g were tested. To determine the effect of contact time and to evaluate adsorption kinetics, experiments were performed at 25 °C using an initial dye concentration of 400 mg L^−1^ and 0.01 g of adsorbent. Contact times of 60, 90, 120, 150, 180, 210, and 240 min were applied. The effect of temperature and the thermodynamic characteristics of the adsorption process were investigated at 25, 35, 45, and 55 °C, while maintaining an initial dye concentration of 400 mg L^−1^, an adsorbent dose of 0.01 g, and a contact time of 120 min. To evaluate the influence of initial dye concentration and to establish adsorption isotherms, dye solutions were prepared in the concentration range of 25–750 mg L^−1^. These experiments were carried out at 25 °C with 0.01 g of adsorbent and a contact time of 120 min. In each experiment, 30 mL of dye solution at the desired concentration was brought into contact with the specified amount of Ba_2_Mg(BO_3_)_2_ under controlled temperature and time conditions. All experiments were conducted in reactor vessels using an orbital shaker operated at 300 rpm to ensure homogeneous mixing. Dye solutions (30 mL) at the specified concentrations (concentration experiments: 25–750 mg L^−1^; other parameter studies: 400 mg L^−1^) were contacted with the specified amounts of adsorbent (dosage experiments: 0.005–0.02 g; other parameter studies: 0.01 g), after which the solution and adsorbent were separated using filter paper. The remaining solution was diluted to 50 mL in volumetric flasks, and the residual dye concentration was determined using a UV-vis spectrophotometer. The equilibrium adsorption capacity of the dyes was calculated using eqn (1), after applying the corresponding dilution factors.1
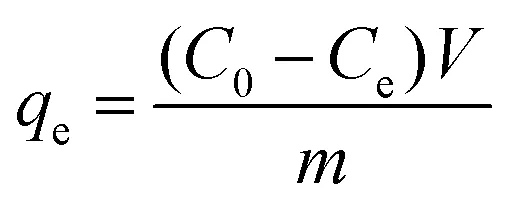
where: *C*_0_ is the initial concentration of MG and CRV, *C*_e_ is the equilibrium concentration, *m* (g) is the dry mass of Ba_2_Mg(BO_3_)_2_ used as the adsorbent and *V* (L) is the solution volume. The R programming language and RStudio software were used for data analysis and graph plotting.^48,49^ For the temperature dependent analysis, a dimensionless equilibrium ratio derived from the initial and equilibrium dye concentrations was used in the thermodynamic evaluation. The temperature dependence of the process was assessed from the linear relationship between ln *K* and 1/*T*.

## Results and discussion

### FT-IR analysis of Ba_2_Mg(BO_3_)_2_

The chemical bonding structure and functional groups of the Ba_2_Mg(BO_3_)_2_ adsorbent were analyzed using FT-IR spectroscopy. Examination of the FT-IR spectrum presented in Fig. 1 indicates the presence of characteristic vibrational bands associated with the metal borate structure. The absorption band at 1190 cm^−1^ is assigned to the B–O stretching vibrations of trigonal BO_3_ units. The bands at approximately 844 and 857 cm^−1^ are associated with B–O bending vibrations of BO_3_ units. These bands support the presence of trigonal BO_3_ units in the structure.^50,51^ The bands at 693 and 589 cm^−1^ may be attributed to BO_3_ bending and metal oxygen lattice vibrations, respectively. The band at approximately 1419 cm^−1^ is attributed to asymmetric B–O stretching of trigonal BO_3_ units.

**Fig. 1:**
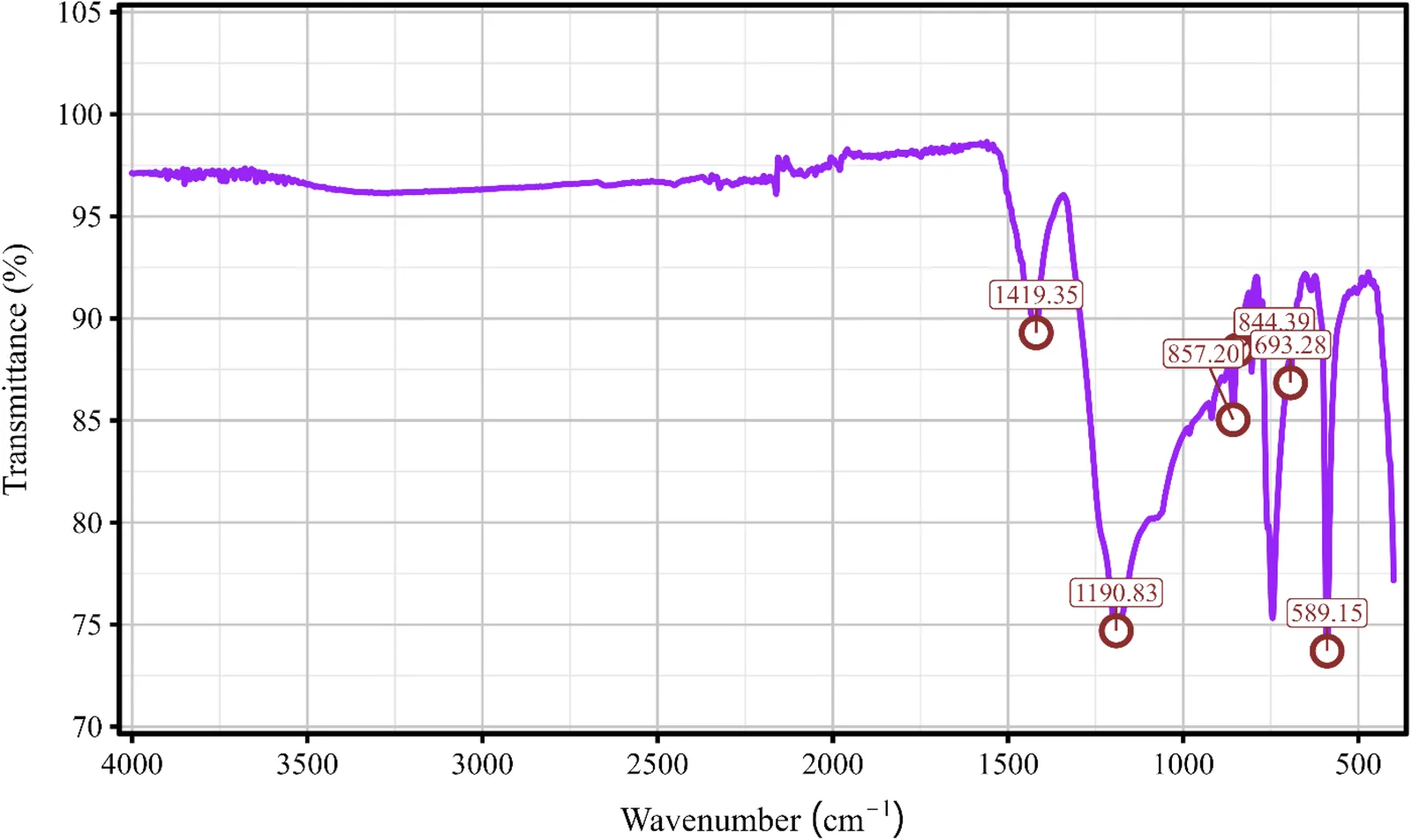
FT-IR spectrum of Ba_2_Mg(BO_3_)_2_ indicating characteristic vibrational bands.

### Morphology and elemental composition of Ba_2_Mg(BO_3_)_2_

The surface morphology and elemental composition of the synthesized Ba_2_Mg(BO_3_)_2_ adsorbent were examined in detail using SEM images obtained at different magnifications and EDS analysis. The SEM micrographs presented in Fig. 2a–f indicate that the surface of Ba_2_Mg(BO_3_)_2_ exhibits a heterogeneous and irregular morphology. Fig. 2a and b show that irregularly shaped particles are aggregated, forming relatively large agglomerates. This structural feature may be attributed to the fusion of metal borate phases formed during the synthesis process, resulting in a compact architecture. The porous and irregular surface texture may facilitate the binding of dye molecules to the adsorbent surface. The EDS spectrum (Fig. 2g) supports the presence of Ba, Mg, B, and O in the analyzed region. The C signal is mainly attributed to the carbon adhesive tape used for sample mounting.

**Fig. 2:**
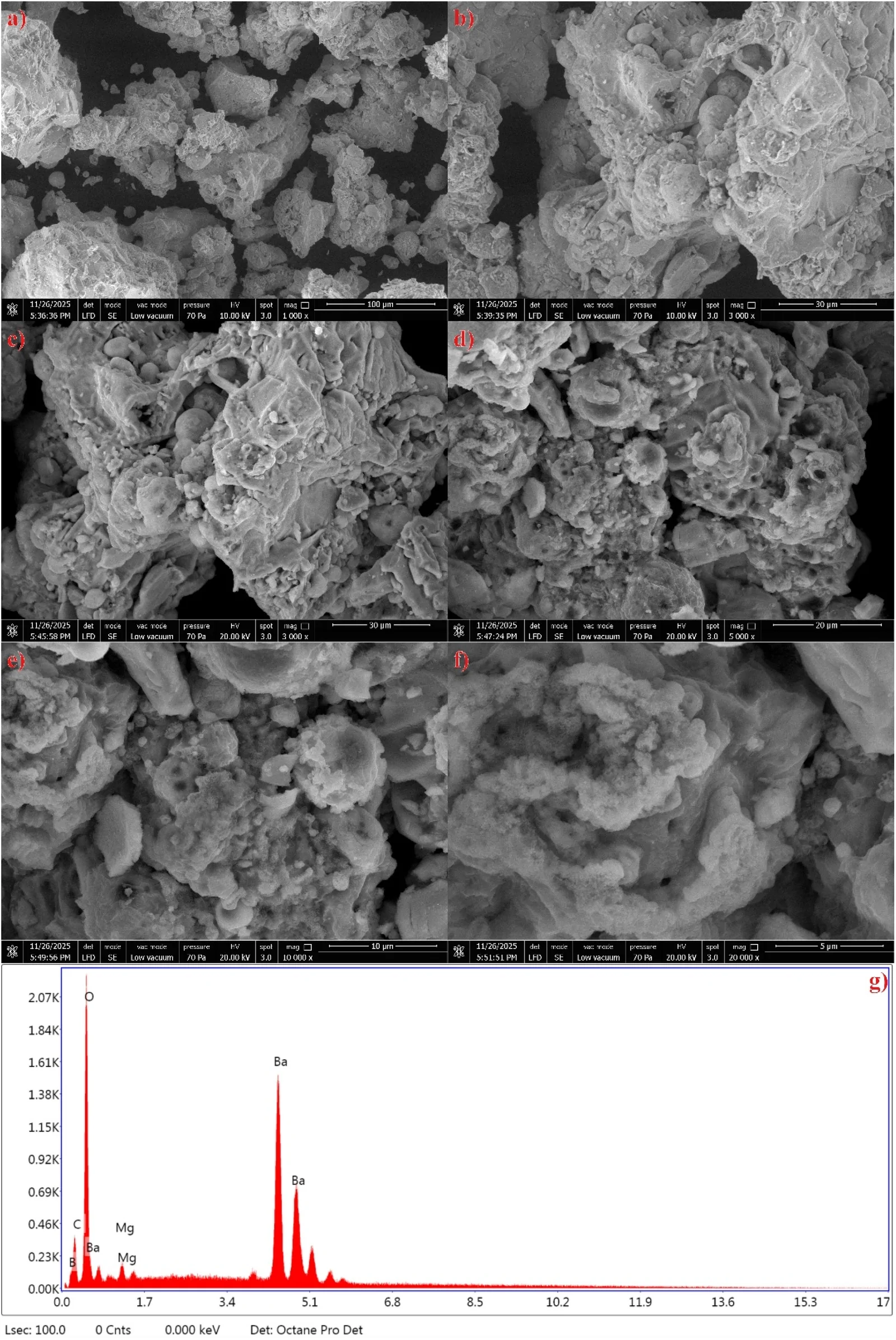
SEM micrographs and EDS spectrum of Ba_2_Mg(BO_3_)_2_ at different magnifications (a) 1000×, (b) 3000×, (c) 3000×, (d) 5000×, (e) 100 00×, (f) 200 00×, (g) EDS spectrum.

### Surface area and pore characteristics of Ba_2_Mg(BO_3_)_2_

BET surface area is determined by gas adsorption measurements, typically using N_2_ at 77 K under cryogenic conditions, and is widely applied for the characterization of porous solids and fine powders.^52^ The nitrogen adsorption–desorption isotherm obtained for Ba_2_Mg(BO_3_)_2_ is presented in Fig. 3. According to the BET analysis, the specific surface area of Ba_2_Mg(BO_3_)_2_ was determined to be 1.3244 m^2^ g^−1^. The micropore surface area and external surface area calculated by the *t*-plot method were 0.1022 and 1.2221 m^2^ g^−1^, respectively, indicating that microporosity contributes a small fraction of the total surface area. The total pore volume determined at a relative pressure of approximately 0.995 was 0.011766 cm^3^ g^−1^, while the *t*-plot micropore volume was 0.000030 cm^3^ g^−1^. The average pore diameter calculated by the BET 4 V/A method was 355.362 Å, whereas the BJH adsorption average pore width was 443.461 Å. The BJH pore size distribution spanned both the mesopore and macropore ranges, indicating a broad pore size distribution.

**Fig. 3:**
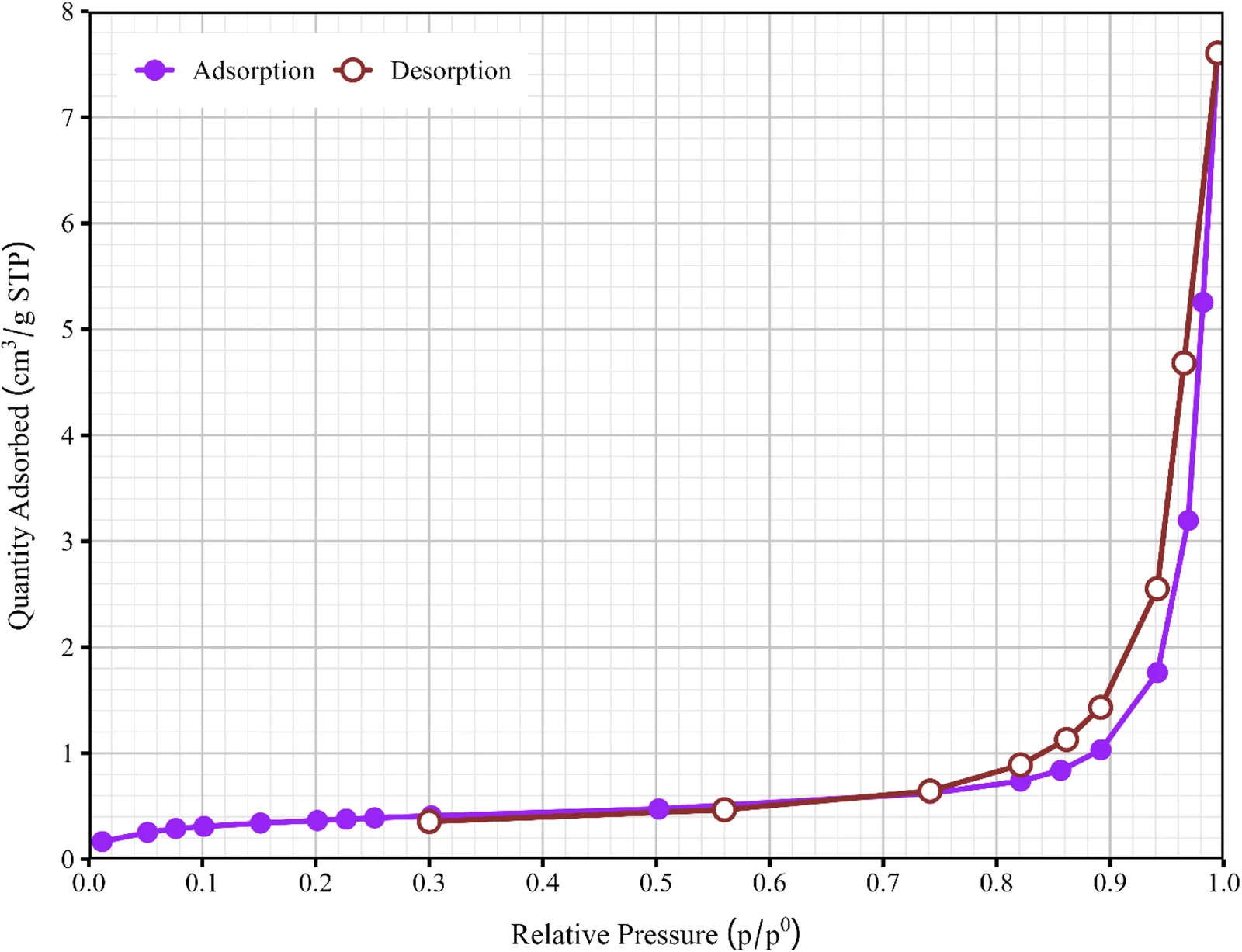
N_2_ adsorption–desorption isotherm and pore structure characteristics of Ba_2_Mg(BO_3_)_2_.

In a study conducted by Mahmoodi-Babolan *et al.* (2019),^53^ the specific surface area and adsorption capacity of a adsorbent used for methylene blue removal were reported as 2.8031 m^2^ g^−1^ and 2276 mg g^−1^, respectively. Naim *et al.* (2022)^54^ reported that the Amberlite IRA 958-Cl material exhibited a BET surface area of 2.03 m^2^ g^−1^ and a sorption capacity of 537.63 mg g^−1^. In another study, Rahmati *et al.* (2026)^55^ found that the synthesized material (Al-CP) had a BET surface area of 4.46 m^2^ g^−1^ and an adsorption capacity ranging from 1456.5 to 2077.5 mg g^−1^. Similarly, Abdulhameed *et al.* (2026)^56^ reported an adsorption capacity of 316.96 mg g^−1^ and a BET surface area of 9.42 m^2^ g^−1^ for the CTO/PC-HA material. In contrast, Ebrahim *et al.* (2026)^57^ observed that the PEA@ZIF-67 material possessed a significantly higher BET surface area of 145.3 m^2^ g^−1^ and an adsorption capacity of 4348 mg g^−1^. These studies suggest that materials with relatively low surface areas can still exhibit high adsorption capacities. Conversely, Alsowayigh *et al.* (2026)^58^ reported a material with a BET surface area of 118.6 m^2^ g^−1^ but a comparatively low adsorption capacity of 31.2 mg g^−1^. In a study by Goswami and Dey (2022),^59^ the BET surface area of the ACMAS adsorbent used in CRV dye removal was found to be 191.25 m^2^ g^−1^ before adsorption and 202.64 m^2^ g^−1^ after adsorption, and the maximum adsorption capacity was determined as 235.7 mg g^−1^. Overall, these findings indicate that there is not always a direct positive correlation between BET surface area and adsorption capacity. Materials with low surface area may demonstrate high adsorption performance, while materials with relatively high surface area may exhibit lower adsorption capacities, depending on factors such as surface chemistry, active site density, and interaction mechanisms.

### Crystal structure of Ba_2_Mg(BO_3_)_2_

The XRD pattern presented in Fig. 4 indicates the crystalline nature of the Ba_2_Mg(BO_3_)_2_ material synthesized by the solution combustion method. The sharp diffraction peaks support the formation of a crystalline phase. The peaks corresponding to the main reflections were identified at approximately 2*θ* = 16.08°, 19.91°, 22.00°, 28.96°, 32.49°, 33.29°, 33.52°, 40.46°, 44.87°, 53.51°, 57.15°, 59.78°, 61.22°, and 70.44°. These peaks can be assigned to the (003), (101), (012), (104), (006), (015), (110), (202), (024), (122), (214), (125), (119), and (128) planes of the Ba_2_Mg(BO_3_)_2_ phase, respectively. Comparison with the standard card data shows that the experimental peak positions are consistent with the PDF 01-082-1883/JCPDS 82-1883 card reported for Ba_2_Mg(BO_3_)_2_. In particular, the clear observation of the (012), (104), (015), (110), (202), and (024) planes, which are among the most intense reference reflections, in the experimental pattern supports the successful formation of the barium magnesium borate phase. No additional crystalline impurity peaks were detected within the detection limits of the XRD analysis.^60–63^

**Fig. 4:**
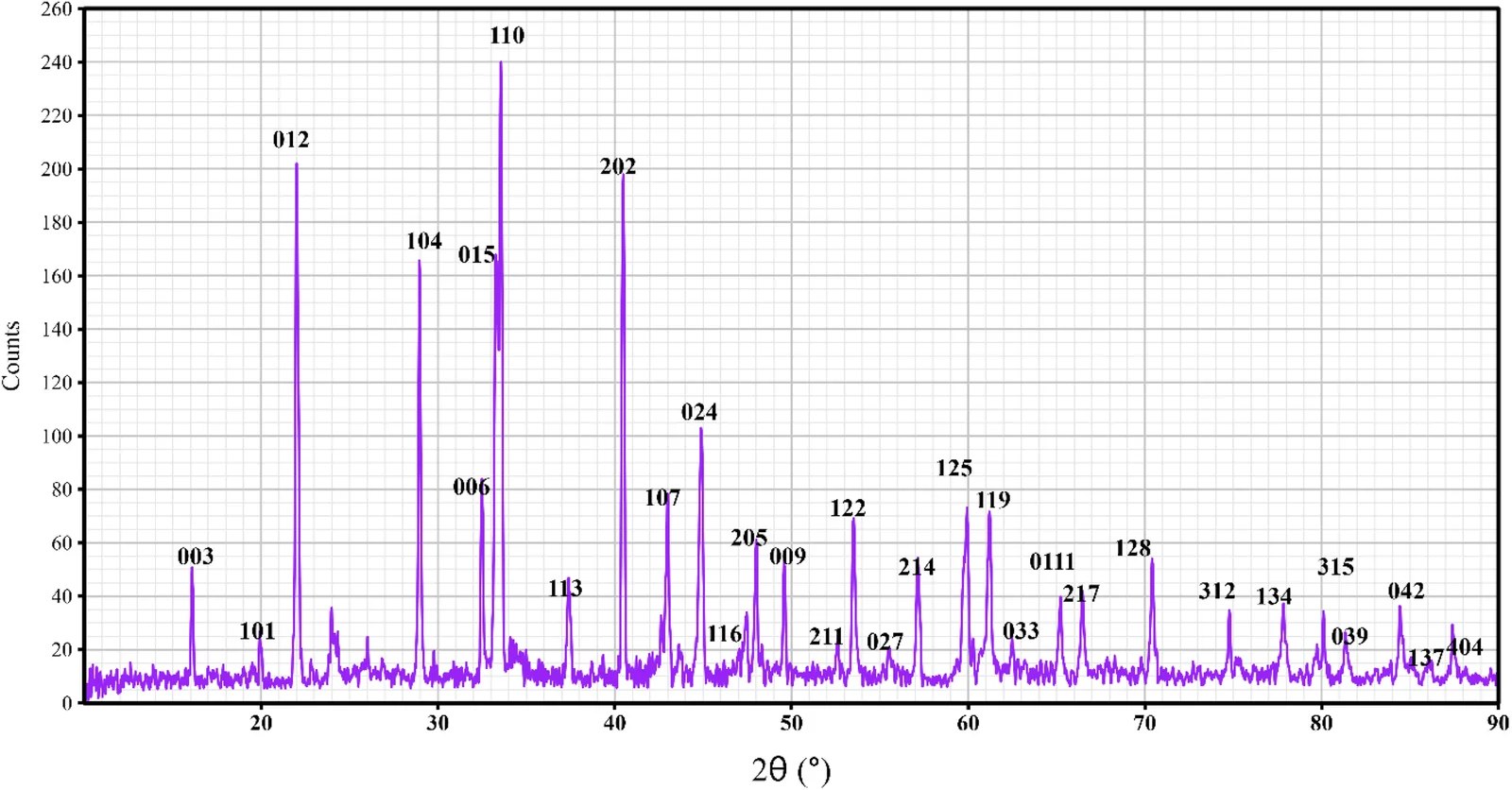
The XRD pattern of Ba_2_Mg(BO_3_)_2_ prepared by the solution combustion method.

In the literature, the Ba_2_Mg(BO_3_)_2_ structure has been reported to be a buetschliite-derived orthoborate phase that crystallizes in the *R*3̄*m* space group in the trigonal/rhombohedral hexagonal axis setting. According to the crystal structure data, the unit cell parameters are given as approximately *a* = 5.343 Å, *c* = 16.520 Å, and *Z* = 3. In the structure, planar BO_3_ groups form layers perpendicular to the crystallographic *c*-axis, while layers composed of Ba and Mg atoms are located between these layers. Mg atoms are situated within slightly distorted octahedral MgO_6_ environments, whereas Ba atoms possess irregular, anisotropic nine-coordinate oxygen environments. These crystal structure characteristics lead to the appearance of numerous sharp reflections in the XRD pattern and indicate that the obtained pattern is consistent with the Ba_2_Mg(BO_3_)_2_ orthoborate structure.^43–45,60–63^ Consequently, the XRD pattern of the synthesized sample shows a high degree of agreement with the Ba_2_Mg(BO_3_)_2_ crystal structure reported in the literature and the standard PDF 01-082-1883 card. These results confirm that the diffraction pattern is consistent with the reported Ba_2_Mg(BO_3_)_2_ orthoborate structure.^43–45^

### Particle size distribution of Ba_2_Mg(BO_3_)_2_

The particle size distribution of the synthesized material is presented in Fig. 5. The median particle diameter (*D*50) was 48.20 µm. The volume-weighted mean diameter (*D*[4,3]) was 54.64 µm, whereas the surface area weighted mean diameter (*D*[3,2]) was 7.228 µm. The pronounced difference between *D*[4,3] and *D*[3,2] indicates the presence of a fine fraction that contributes significantly to the overall surface area of the system. The *D*10 and *D*90 values were 9.221 and 106.6 µm, respectively. Together with a SPAN value of 2.02, this wide range demonstrates that the sample has a polydisperse and heterogeneous particle size distribution. The *D*90/*D*10 ratio of approximately 11.56 further confirms substantial particle size heterogeneity within the system. Such a broad size distribution may influence the adsorption process by promoting multistep mass transfer behavior.

**Fig. 5:**
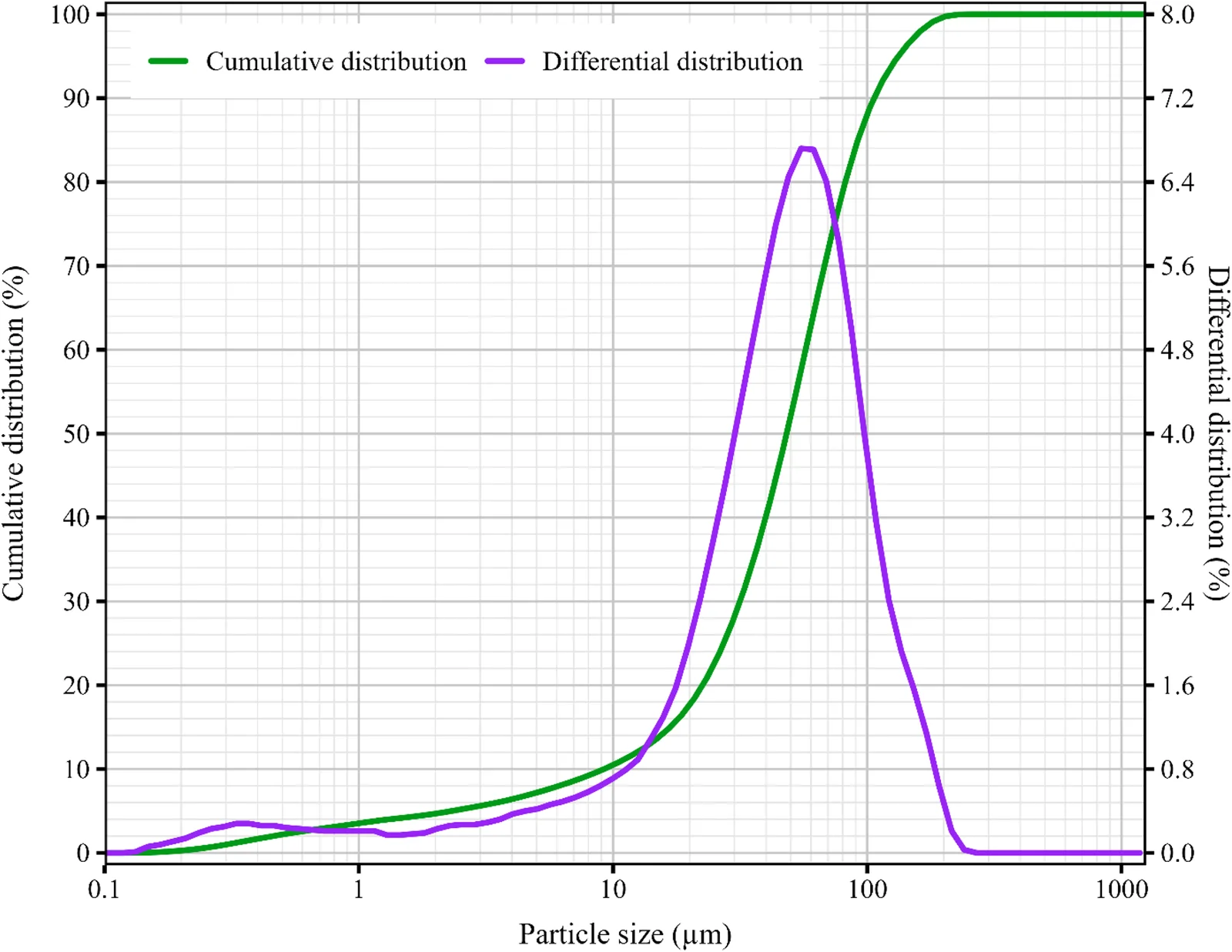
Particle size distribution profile of Ba_2_Mg(BO_3_)_2_.

### Thermal stability of Ba_2_Mg(BO_3_)_2_

The thermal stability of the Ba_2_Mg(BO_3_)_2_ adsorbent was evaluated by thermogravimetric (TG) and differential thermogravimetric (DTG) analyses over a wide temperature range, as shown in Fig. 6. Examination of the TG curve indicates that the total mass loss of the sample within the 25–1400 °C range is very limited. Although minor signals are observed in the DTG curve at temperatures above 1000 °C, the corresponding changes in the TG profile remain relatively small (approximately 0.1–0.4%). These features may be associated with minor thermal events. As shown in Fig. 6, the total mass loss at 1400 °C is approximately 3%, corresponding to the retention of approximately 97% of the initial sample mass. These results demonstrate the high thermal stability of Ba_2_Mg(BO_3_)_2_ in terms of mass retention over the investigated temperature range.

**Fig. 6:**
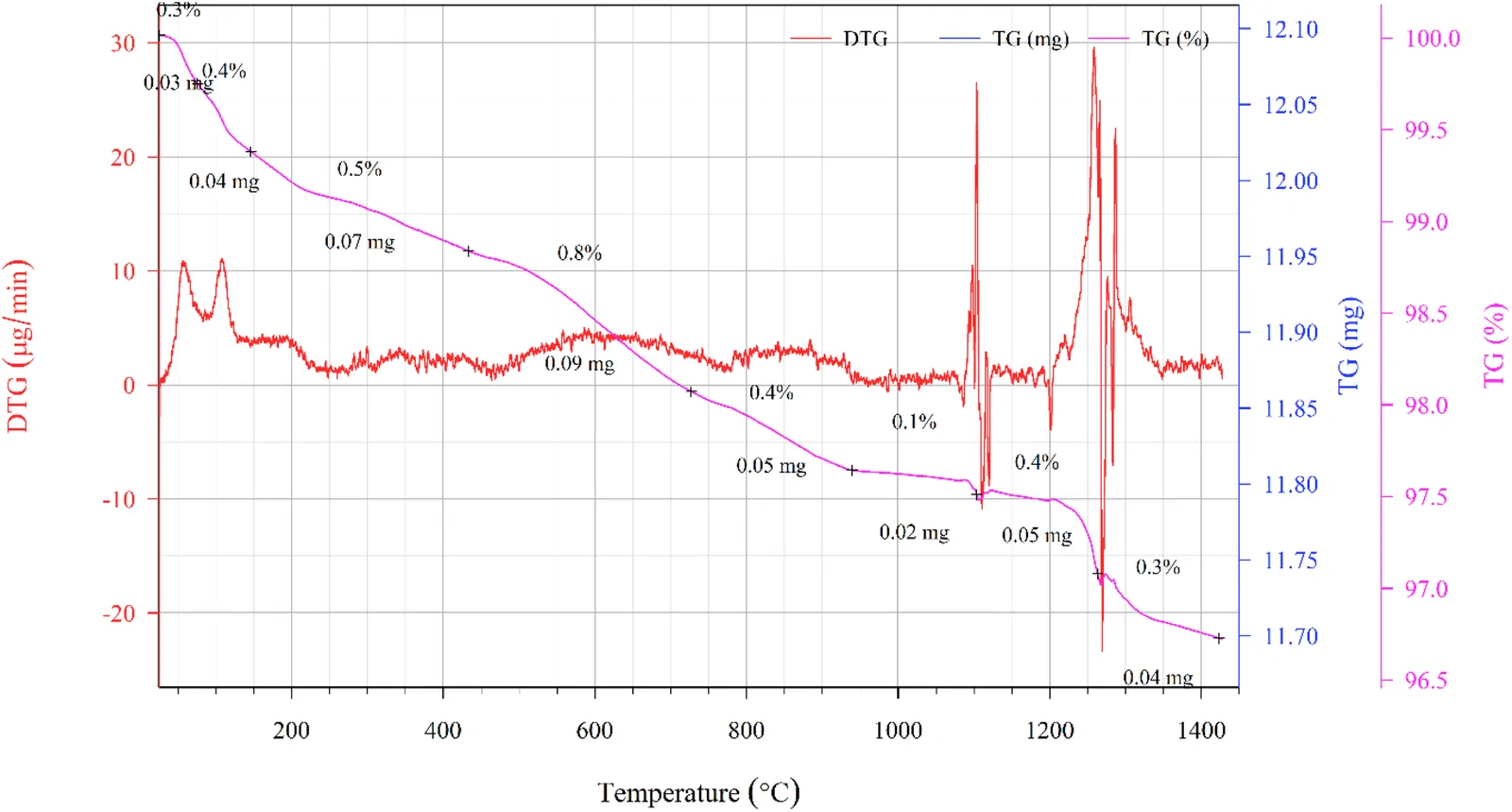
TG-DTG curves of Ba_2_Mg(BO_3_)_2_ demonstrating thermal stability.

### Surface charge characteristics and effect of solution pH

Zeta potential analysis was performed to evaluate the colloidal behavior and surface charge characteristics of Ba_2_Mg(BO_3_)_2_. The distribution profile shown in Fig. 7 exhibits a single, narrow peak centered at approximately −50.5 mV, indicating a relatively uniform surface charge distribution within the system. The narrow distribution indicates relatively narrow electrophoretic mobility. The negative zeta potential may indicates negatively charged surface under the measurement conditions. This surface charge may favor electrostatic interactions with the cationic dye molecules.

**Fig. 7:**
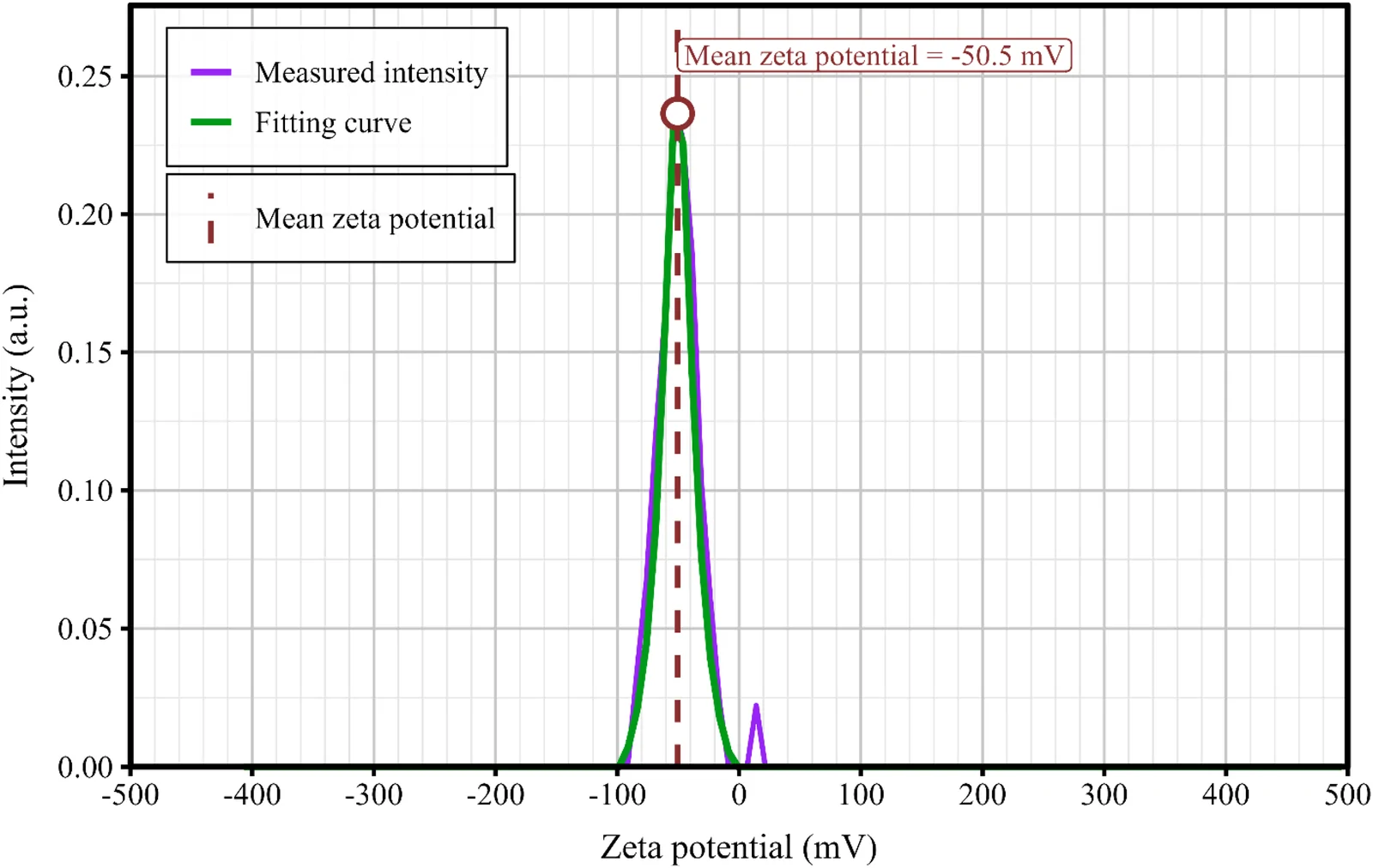
Zeta potential distribution of Ba_2_Mg(BO_3_)_2_.

Fig. 8 illustrates the effect of pH, as one of the key adsorption parameters, on the removal performance of MG and CRV by Ba_2_Mg(BO_3_)_2_. The results indicate that the adsorption efficiency for both dyes increases significantly with increasing pH and reaches maximum values under slightly alkaline and alkaline conditions. For MG, the adsorption efficiency was 74.272% at pH 4.5. This value increased progressively with rising pH and exceeded 99% from pH 7.5 onward. The removal efficiency remained nearly constant in the pH range of 7.5–10.5, varying between approximately 99.475% and 99.486%. This indicates that further increases in pH had a limited effect on the removal performance within this interval. A study by Shakeel *et al.* (2025)^64^ also indicated that maximum degradation occurred at pH 8.5 in MG removal, after which equilibrium was reached. Similarly, for CRV, the adsorption efficiency was 81.690% at pH 4.5 and increased with increasing pH, surpassing 94.489% from pH 6.5 onward. Within the pH range of 7.5–10.5, the removal efficiency for CRV varied between 99.589% and 99.735%, indicating consistently high adsorption performance under alkaline conditions. The removal at higher pH may be related to changes in the surface charge of adsorbent.^65^ This may enhance electrostatic interactions between the surface and the cationic MG and CRV molecules. A comparison of the adsorption behaviors of MG and CRV reveals that CRV exhibited higher removal efficiency than MG, particularly under acidic conditions. However, under alkaline conditions, pH induced changes that may occur in the spectral response of MG should also be taken into consideration.

**Fig. 8:**
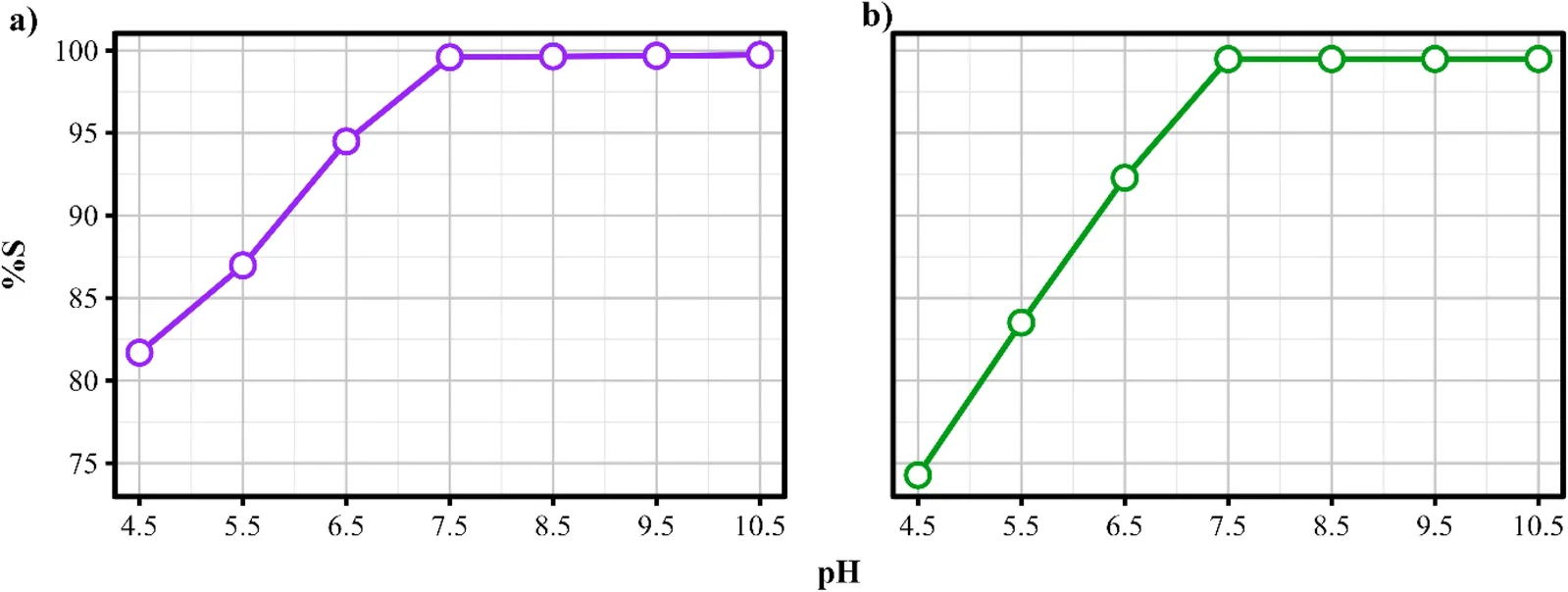
Effect of initial solution pH on the adsorption efficiency of (a) CRV and (b) MG onto Ba_2_Mg(BO_3_)_2_ (conditions; concentration: 400 mg L^−1^–30 mL dye, dosage: 0.01 g, temperature: 25 °C, time: 120 min, pH: 4.5–10.5).

### Effect of adsorbent dosage on adsorption performance

The effect of Ba_2_Mg(BO_3_)_2_ adsorbent dosage on the removal efficiency of the cationic dyes is presented separately for MG and CRV in Fig. 9, within the dosage range of 0.005–0.02 g. The results indicate that, for both dyes, the adsorption efficiency increases markedly with increasing adsorbent dosage and reaches a plateau beyond a certain amount. For MG, the removal efficiency was 82.757% at an adsorbent dose of 0.005 g. Upon increasing the adsorbent amount to 0.01 g, the removal efficiency reached 99.475% and only marginal changes were observed at higher dosages (0.015 g and 0.02 g), where the values remained nearly constant. A similar trend was observed for CRV. At 0.005 g of adsorbent, the removal efficiency was 87.433%. When the adsorbent dose reached 0.01 g, the removal efficiency increased to 99.589% and remained essentially unchanged at higher dosages (99.616–99.627%). Overall, Ba_2_Mg(BO_3_)_2_ demonstrates high adsorption efficiency toward both cationic dyes.

**Fig. 9:**
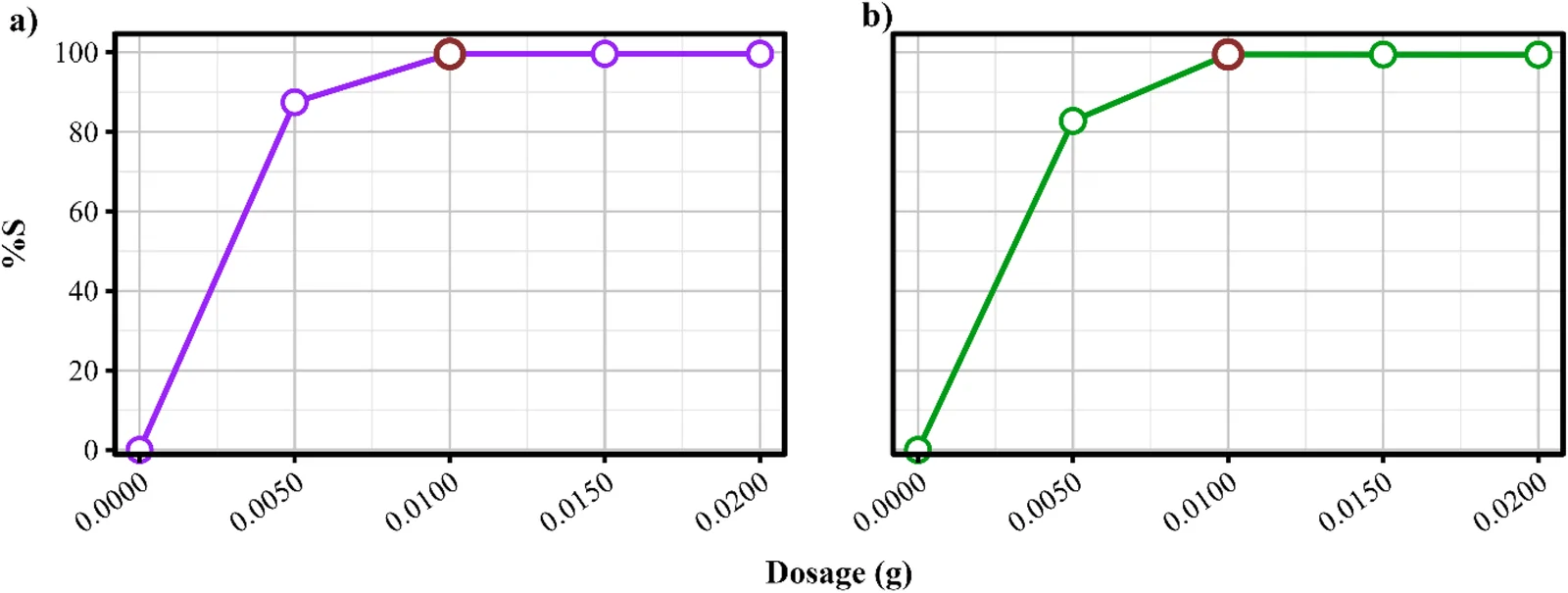
Effect of adsorbent dosage on the adsorption efficiency of (a) CRV and (b) MG onto Ba_2_Mg(BO_3_)_2_ (conditions; concentration: 400 mg L^−1^–30 mL dye, dosage: 0.005 g, 0.01 g, 0.015 g, 0.02 g, temperature: 25 °C, time: 120 min, pH: natural).

### Effect of contact time and kinetic analysis

Contact time is known to be an important factor affecting the removal efficiency of adsorbent materials.^66^Fig. 10 illustrates the effect of contact time on the removal of MG and CRV by Ba_2_Mg(BO_3_)_2_ over the range of 60–240 min. For MG, the adsorption efficiency was 90.162% at 60 min and increased significantly with increasing contact time. The removal efficiency reached 94.045% at 90 min and 99.475% at 120 min. Within the 150–240 minute interval, the MG removal efficiency varied between 99.53% and 99.81%, indicating that adsorption equilibrium had been achieved. In the case of CRV, a relatively high removal efficiency of 94.663% was observed even at 60 min. The efficiency increased to 97.365% at 90 min and reached 99.589% at 120 min. CRV exhibited higher removal efficiencies than MG during the early stage of adsorption, whereas both dyes approached a plateau at approximately 120 min. A study by Goswami and Dey (2022)^67^ also yielded similar results, showing that the removal capacity of SLS-AC adsorbent for MG dye removal increased with increasing contact time and approached a plateau at the reported equilibrium time.

**Fig. 10:**
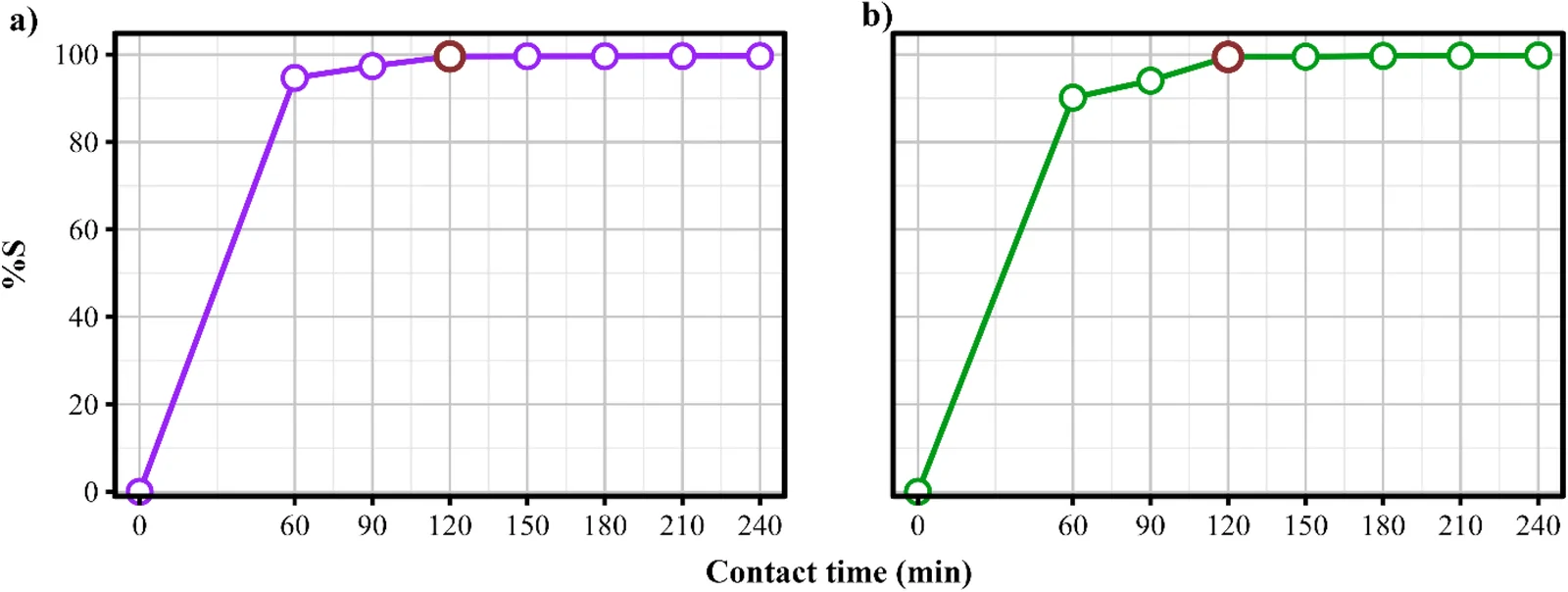
Effect of contact time on the adsorption efficiency of (a) CRV and (b) MG onto Ba_2_Mg(BO_3_)_2_ (conditions; concentration: 400 mg L^−1^–30 mL dye, dosage: 0.01 g, temperature: 25 °C, time: 60, 90, 120, 150, 180, 210, 240 min, pH: natural).

The adsorption kinetics of MG and CRV onto Ba_2_Mg(BO_3_)_2_ were investigated using the pseudo-first-order (PFO), pseudo-second-order (PSO) and intra-particle diffusion models. The calculated kinetic parameters are presented in Table 1 and Fig. 11. For MG, the PSO model exhibited a high correlation coefficient (*R*^2^ = 0.999). The equilibrium adsorption capacity calculated from the PSO model (*q*_e,cal_ = 1242.476 mg g^−1^) was reasonably close to the experimental value (*q*_e,exp_ = 1197.774 mg g^−1^). Similarly, in the removal of CRV by Ba_2_Mg(BO_3_)_2_, the PSO model demonstrated an excellent fit (*R*^2^ = 0.999). The equilibrium adsorption capacity calculated from the PSO model (*q*_e,cal_ = 1216.048 mg g^−1^) was reasonably close to the experimental value (*q*_e,exp_ = 1196.109 mg g^−1^). The PSO model provided the best empirical fit among the investigated kinetic models for MG and CRV.

**Table 1:** Kinetic model parameters for MG and CRV adsorption onto Ba_2_Mg(BO_3_)_2_

Dyes	Model	*q*_e,exp_	*q*_e,cal_	*k*_1_/*k*_2_/*k*_id_	*C* (mg g^−1^)	*R* ^2^
CRV	PFO	1196.109	512.081	0.041 min^−1^		0.896
	PSO	1196.109	1216.048	2.480 × 10^−4^ g mg^−1^ min^−1^		0.999
	Intraparticle	1196.109		7.030 mg g^−1^ min^−1/2^	1099.011	0.705
MG	PFO	1197.774	783.262	0.035 min^−1^		0.906
	PSO	1197.774	1242.476	1.067 × 10^−4^ g mg^−1^ min^−1^		0.999
	Intraparticle	1197.774		14.390 mg g^−1^ min^−1/2^	997.609	0.743

**Fig. 11:**
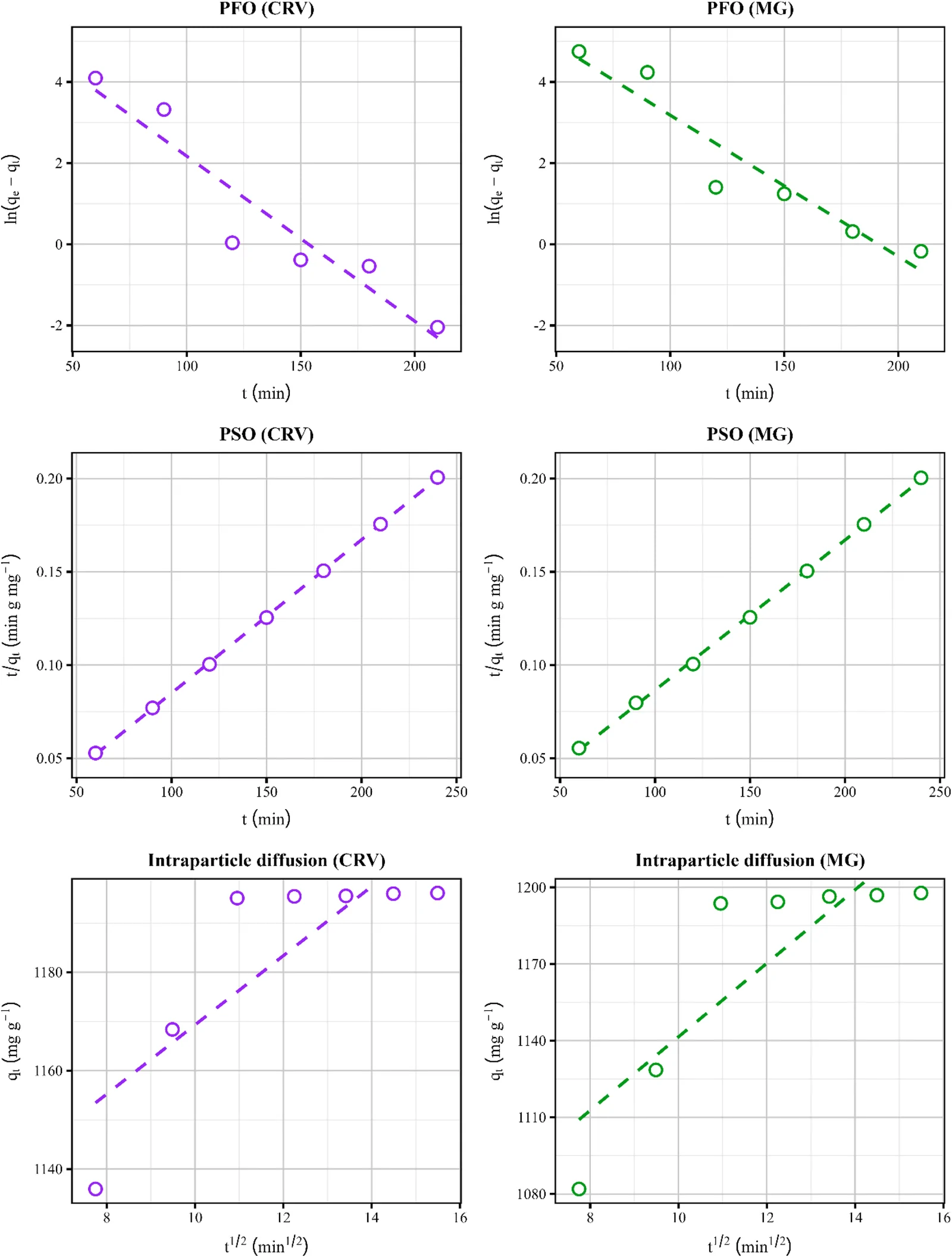
Kinetic model fitting plots for MG and CRV adsorption onto Ba_2_Mg(BO_3_)_2_.

### Effect of temperature and thermodynamic analysis

The effect of temperature on MG and CRV adsorption was investigated over the range of 25–55 °C, as shown in Fig. 12. The adsorption efficiency of both dyes increased slightly with temperature. For MG, the removal efficiency increased from 99.48% at 25 °C to 99.72% at 55 °C. CRV showed a similar trend, increasing from 99.59% at 25 °C to 99.73% at 55 °C. The slight improvement in removal performance with increasing temperature may be associated with the increased kinetic energy of the dye molecules, which may facilitate their diffusion from the solution to the adsorbent surface. However, the relatively small change in removal efficiency suggests that temperature has a limited but supportive influence on the overall adsorption performance. The results also imply that the adsorption process is primarily governed by surface interactions and remains stable over the investigated temperature range.

**Fig. 12:**
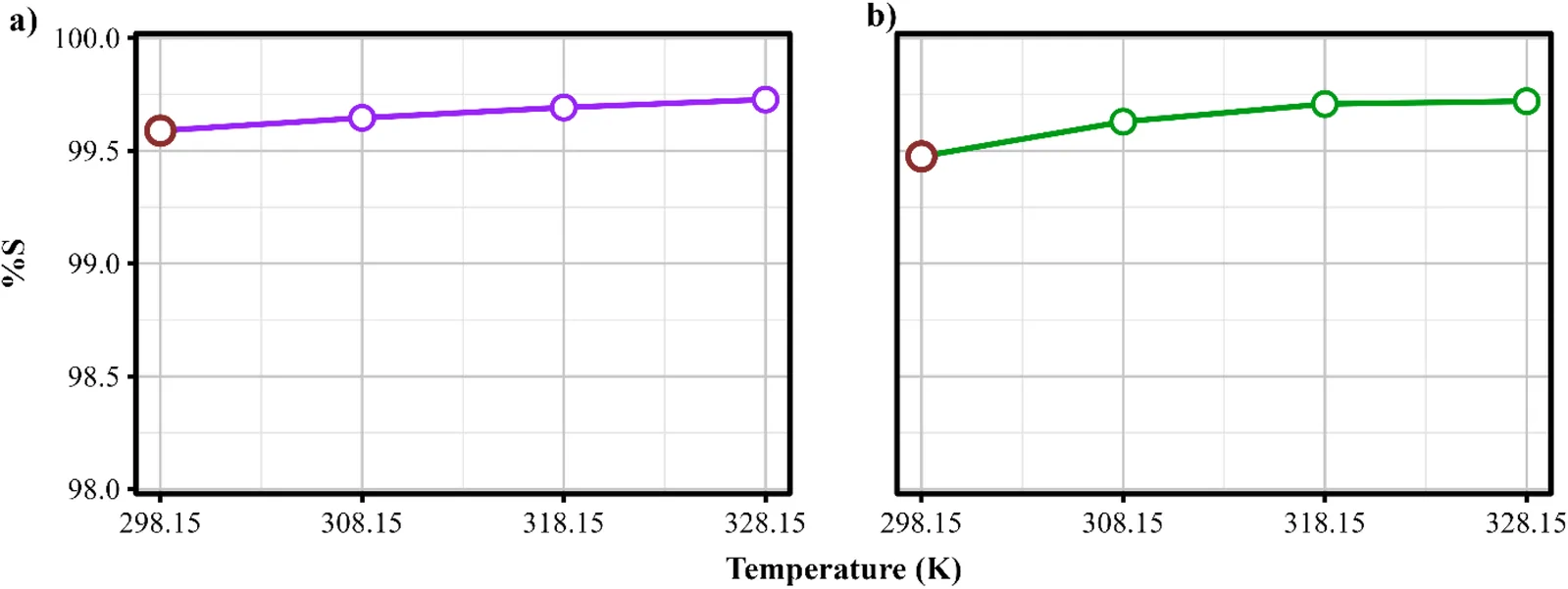
Effect of temperature on the adsorption efficiency of (a) CRV and (b) MG onto Ba_2_Mg(BO_3_)_2_ (conditions; concentration: 400 mg L^−1^–30 mL dye, dosage: 0.01 g, temperature: (25 °C = 298.15 K, 35 °C = 308.15 K, 45 °C = 318.15 K, 55 °C = 328.15 K), time: 120 min, pH: natural).

The thermodynamic characteristics of MG and CRV removal by Ba_2_Mg(BO_3_)_2_ were evaluated using the Gibbs free energy (Δ*G*), enthalpy (Δ*H*), and entropy (Δ*S*) parameters calculated at different temperatures. The thermodynamic parameters are presented in Table 2. For MG, Δ*G* ranged from −13.156 to −16.236 kJ mol^−1^ over the temperature interval of 298.15–328.15 K. The negative Δ*G* values at all investigated temperatures indicate that adsorption occurs spontaneously. The increasingly negative values with rising temperature also suggest that adsorption becomes more favorable at higher temperatures, supporting the positive influence of temperature on the process. CRV showed a similar trend, with Δ*G* values ranging from −13.612 to −16.100 kJ mol^−1^. These results suggest that the adsorption of both MG and CRV onto Ba_2_Mg(BO_3_)_2_ is thermodynamically spontaneous. The positive Δ*H* values of 17.45 kJ mol^−1^ for MG and 11.15 kJ mol^−1^ for CRV indicate that the adsorption process is endothermic. Positive Δ*H* values indicate the endothermic nature of the adsorption process. The positive Δ*S* values of 102.659 J mol^−1^ K^−1^ for MG and 83.072 J mol^−1^ K^−1^ for CRV indicate increased randomness at the solid–liquid interface during adsorption. The positive Δ*S* values may be associated with desolvation and the release of interfacial water molecules during adsorption.

**Table 2:** Thermodynamic parameters for MG and CRV adsorption at different temperatures

	*T* (K)	298.150	308.150	318.150	328.150
MG	Δ*G* (kJ mol^−1^)	−13.156	−14.183	−15.210	−16.236
	Δ*S* (J mol^−1^ K^−1^)	102.659			
	Δ*H* (kJ mol^−1^)	17.451			
CRV	Δ*G* (kJ mol^−1^)	−13.612	−14.451	−15.288	−16.100
	Δ*S* (J mol^−1^ K^−1^)	83.072			
	Δ*H* (kJ mol^−1^)	11.151			

The van't Hoff plots (ln *K versus* 1/*T*) presented in Fig. 13 exhibit a linear relationship with a negative slope for both MG and CRV. This behavior supports the endothermic nature of the adsorption process and indicates that adsorption becomes more favorable with increasing temperature.

**Fig. 13:**
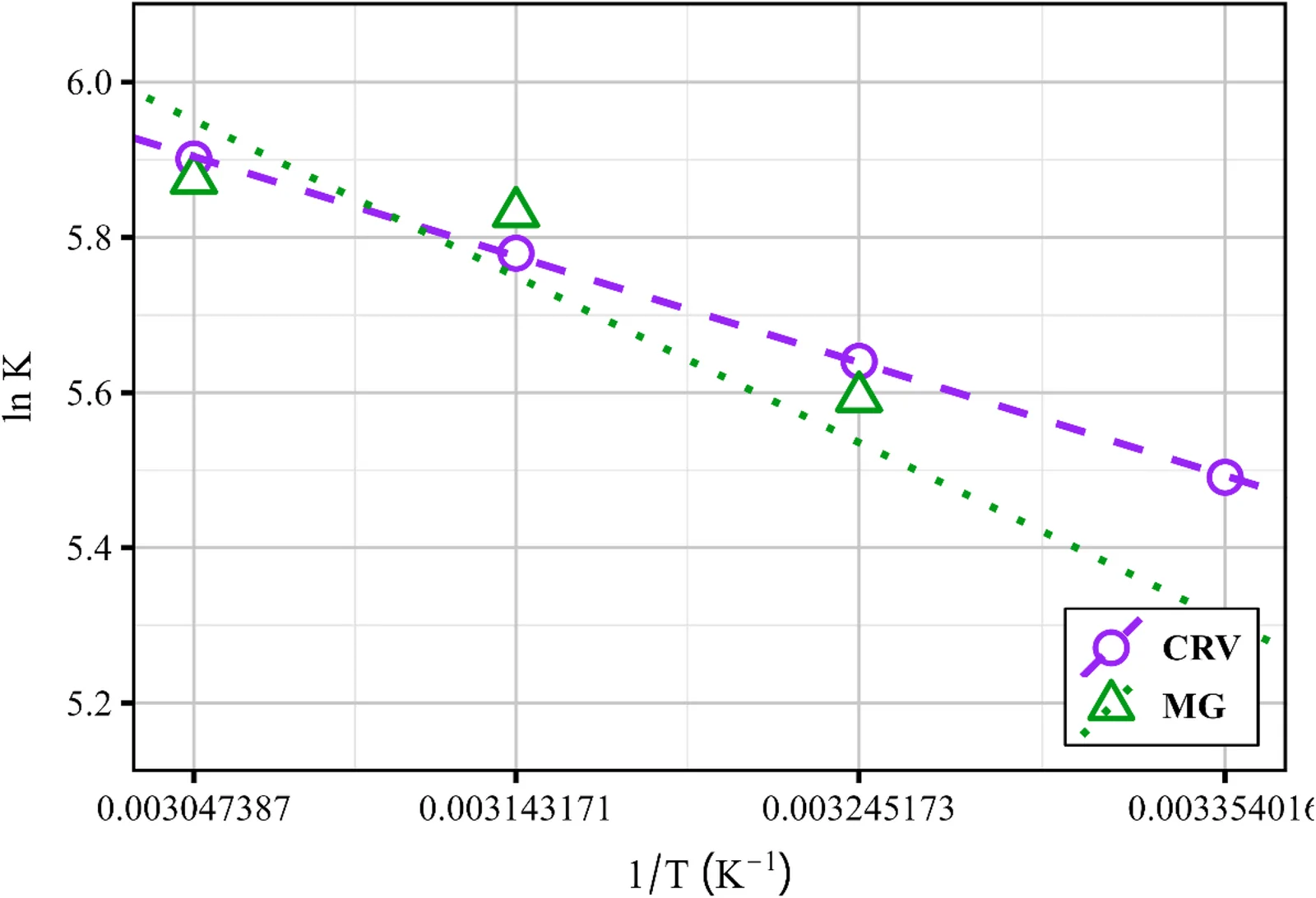
van't Hoff plots (ln *K vs.* 1/*T*) for thermodynamic evaluation.

### Effect of initial dye concentration and isotherm analysis

As shown in Fig. 14, the effect of initial dye concentration on the adsorption capacity of Ba_2_Mg(BO_3_)_2_ was investigated for MG and CRV over the range of 25–750 mg L^−1^. Initial dye concentration is an important parameter for understanding and interpreting the adsorption isotherms of the material. The experimental data were evaluated using the Langmuir,^68^ Freundlich,^69^ Temkin,^70^ and Scatchard,^71^ isotherm models to provide a more detailed assessment of the equilibrium adsorption behavior. The suitability of the models was compared. Comparison of the MG and CRV isotherms showed comparable adsorption capacities for the two dyes over the investigated concentration range.

**Fig. 14:**
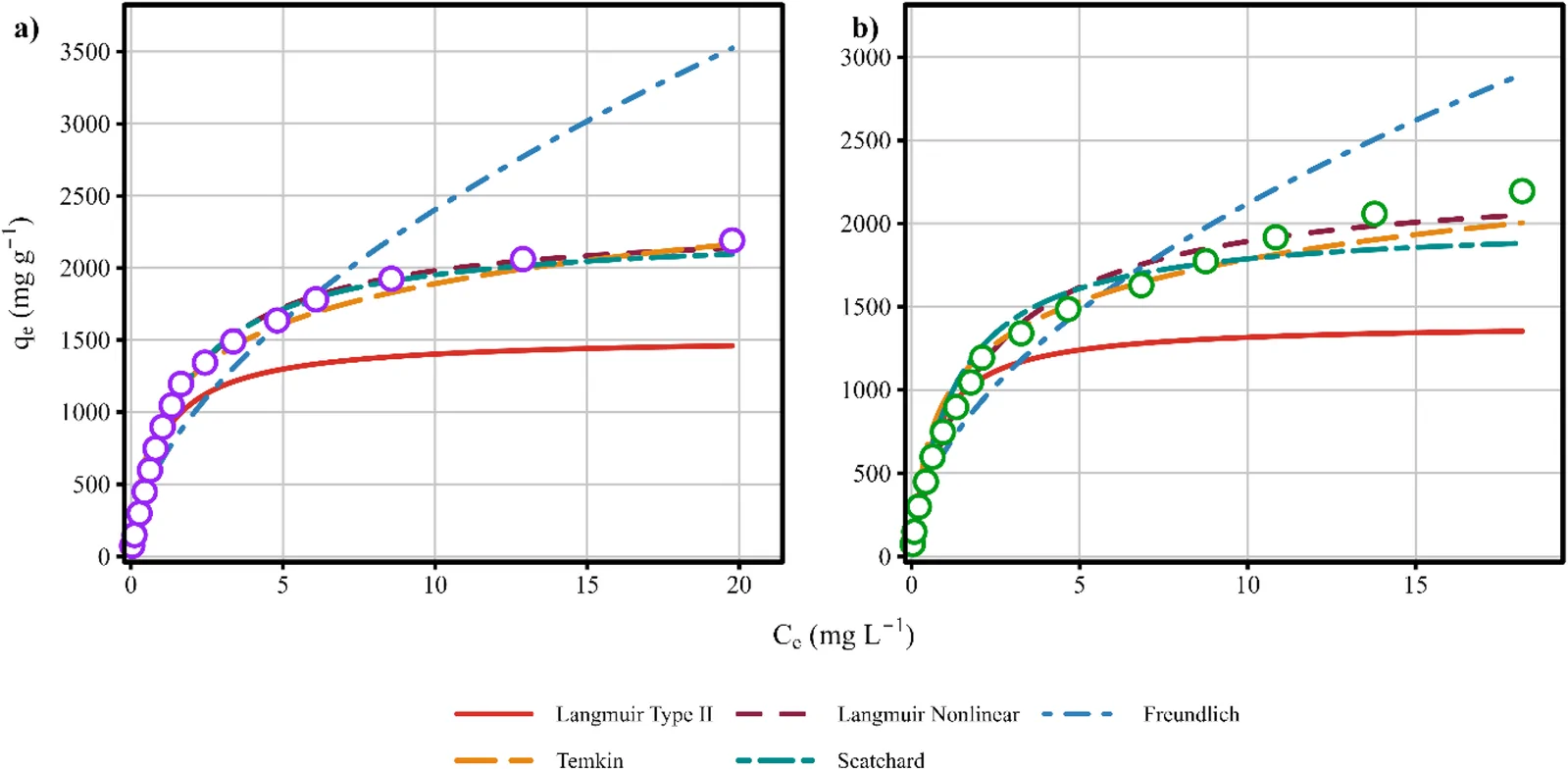
Effect of initial dye concentration on the adsorption capacity of (a) CRV and (b) MG onto Ba_2_Mg(BO_3_)_2_ after 120 min of contact time (conditions; concentration: 25, 50, 100, 150, 200, 250 mg L^−1^, 300, 350, 400, 450, 500, 550, 600, 650, 700, 750 mg L^−1^–30 mL dye, dosage: 0.01 g, temperature: 25 °C, time: 120 min, pH: natural).

The isotherm calculations for MG and CRV removal using Ba_2_Mg(BO_3_)_2_ are presented in Table 3. The results show that the description of MG and CRV adsorption depends on the isotherm model applied. Because the coefficients of determination obtained from linearized transformations are not directly comparable with those calculated on the original *q*_e_ scale, model selection was based primarily on the original-scale *R*^2^ values. For MG, the nonlinear Langmuir model described the experimental data with a high correlation coefficient (*R*^2^ = 0.989). The model predicted a maximum adsorption capacity of 2283.835 mg g^−1^ and a *K*_L_ value of 0.484 L mg^−1^. These results indicate that Ba_2_Mg(BO_3_)_2_ has a high affinity and adsorption capacity toward MG. For CRV, the nonlinear Langmuir model also provided a high fit to the experimental data (*R*^2^ = 0.997). The CRV equilibrium data were well described by the Langmuir-type models within the investigated concentration range. The nonlinear Langmuir model yielded qmax values of 2283.835 and 2327.393 mg g^−1^ for MG and CRV, respectively.

**Table 3:** Isotherm model parameters for MG and CRV adsorption onto Ba_2_Mg(BO_3_)_2_^a^

Isotherm models	Isotherm parameters				RMSE
**Langmuir nonlinear**	***q***_**max**_ **(mg g^−^** ^**1**^ **)**	***K***_**L**_ **(L mg^−^** ^**1**^ **)**	**Original-scale *R*** ^**2**^		
MG	2283.835	0.484	0.989		71.559
CRV	2327.393	0.571	0.997		34.943

**Langmuir type II**	***q***_**max**_ **(mg g^−^** ^**1**^ **)**	***K***_**L**_ **(L mg^−^** ^**1**^ **)**	**Transformed *R*** ^**2**^	**Original-scale *R*** ^**2**^	
MG	1401.185	1.538	0.993	0.712	359.625
CRV	1524.282	1.145	0.985	0.757	330.714

**Freundlich**	***K***_**F**_	**1/*n* (dimensionless)**	**Transformed *R*** ^**2**^	**Original-scale *R*** ^**2**^	
MG	634.898	0.524	0.961	0.858	252.742
CRV	661.128	0.561	0.930	0.622	412.609

**Scatchard/Langmuir type IV**	***q***_**max**_ **(mg g^−^** ^**1**^ **)**	***K***_**L**_ **(L mg^−^** ^**1**^ **)**	**Transformed *R*** ^**2**^	**Original-scale *R*** ^**2**^	
MG	2010.964	0.801	0.865	0.965	125.313
CRV	2266.052	0.621	0.931	0.996	40.580

**Temkin**	***B***_**T**_ **(mg g^−^** ^**1**^ **)**	***K***_**T**_ **(L mg^−^** ^**1**^ **)**	**Original-scale *R*** ^**2**^		
MG	366.435	13.057	0.952		146.695
CRV	400.737	11.189	0.960		134.467

a Langmuir nonlinear *R*^2^: calculated directly on the original *q*_e_ scale, Temkin original-scale *R*^2^: *R*^2^ calculated directly on the original *q*_e_ scale, Langmuir type II Transformed *R*^2^: *R*^2^ on the transformed 1/*q*_e_ scale, Freundlich transformed *R*^2^: *R*^2^ on the transformed ln(*q*_e_) scale, Scatchard transformed *R*^2^: *R*^2^ on the transformed *q*_e_/*C*_e_ scale.

In evaluating the isotherm models, transformed *R*^2^ values were reported to characterize the corresponding linearized representations, whereas original-scale *R*^2^ and RMSE values were used to assess the agreement between the experimental and model predicted *q*_e_ values on the original adsorption capacity scale. Mikolajczyk *et al.* (2024)^72^ reported that the transformations used to linearize the Langmuir equation may alter the regression error structure, produce differences in the fitted parameters and lead to overestimation or underestimation of goodness of fit among different Langmuir linearizations.^72^ They also found that nonlinear regression provided more robust results for the investigated systems and could yield more accurate parameter estimates.^72^ Vitek and Masini (2023)^73^ demonstrated that nonlinear regression directly evaluates all experimental data points and can produce lower deviations from the expected adsorption capacity and affinity parameters than linearized Langmuir equations.^73^ In their study, model fit was also evaluated using *R*^2^ and *χ*^2^ calculated on the original data scale from experimental and calculated *q*_e_ values. The present study likewise showed marked differences between transformed and original-scale *R*^2^ values. Although the transformed *R*^2^ values of the Langmuir Type II model were 0.993 and 0.985 for MG and CRV, respectively, the original-scale *R*^2^ values were 0.712 and 0.757, while the corresponding RMSE values were 359.625 and 330.714, respectively. In contrast, the nonlinear Langmuir model yielded *R*^2^ = 0.989 and RMSE = 71.559 for MG, and *R*^2^ = 0.997 and RMSE = 34.943 for CRV. The Scatchard/Langmuir Type IV model also showed a high original-scale fit, particularly for CRV (*R*^2^ = 0.996, RMSE = 40.580). However, the lower RMSE obtained with the nonlinear Langmuir model indicates a smaller deviation between the experimental *q*_e_ values and the model predictions. Based on these results, the transformed *R*^2^ values may be considered to represent the regression fit in the corresponding linearized coordinates.

Table 4 provides a comparative summary of previously reported studies in which different adsorbents were used for the removal of CRV and MG, along with the corresponding adsorption capacities reported in the literature. Nonlinear Langmuir analysis yielded *q*_max_ values of 2283.835 and 2327.393 mg g^−1^ for MG and CRV, respectively. Despite the relatively low BET surface area of 1.3244 m^2^ g^−1^, high adsorption capacities were observed under the investigated conditions.

**Table 4:** Comparison of adsorption capacities of various adsorbents for MG and CRV removal

Adsorbent	Dye	Reported adsorption capacity (mg g^−1^)	References
ANTs@ZIF-67	CRV	3391.8	74
OPCMA	CRV	58.51	75
Hydrothermal carbonization with pyrolysis (HB-700)	CRV	209	76
Nanomagnetic iron oxide	CRV	16.5	77
Apple stem	CRV	153.85	78
Soybean curd residue	CRV	131.64	79
Lemon peel waste	CRV	71.2	79
Buckwheat	CRV	625	80
Buckwheat/Fe_3_O_4_ (1%)	CRV	680.27	80
MNLFL	CRV	53.47	81
MO_CA-H_3_PO_4__	CRV	469.55	82
CPCM@MXene	CRV	2750	83
Ba_2_Mg(BO_3_)_2_	CRV	2327.393	This study
Ba_2_Mg(BO_3_)_2_	MG	2283.835	This study
mJEB	MG	38.02	84
PBB	MG	13.00	85
nM-PBB	MG	18.22	85
AWPN	MG	377.36	86
MnO_2_-NP-CPC	MG	319.40	87
CNW-*graft*-PAA/LDH	MG	25.7	88
Fe_3_O_4_/rGO	MG	77.73	89
Bentonite	MG	178.60	90

### Proposed adsorption mechanism of MG and CRV onto Ba_2_Mg(BO_3_)_2_

The adsorption of CRV and MG onto Ba_2_Mg(BO_3_)_2_ may involve several concurrent interactions. The negative zeta potential measured for Ba_2_Mg(BO_3_)_2_ under the investigated measurement conditions suggests that electrostatic attraction between the negatively charged surface and the cationic dye molecules may contribute to the removal process. The BO_3_ units, MgO_6_ environments and Ba containing sites may provide additional interaction sites for the dye molecules. van der Waals interactions between the adsorbent surface and the aromatic structures of MG and CRV may provide an additional contribution. Therefore, the removal process may involve several surface interactions acting simultaneously.

Considering the high dye uptake relative to the measured BET surface area, possible contributions from surface associated dye aggregation or adsorbent associated aggregation/flocculation may also be considered. Visible accumulations appeared in some dye solutions after contact between the dye and Ba_2_Mg(BO_3_)_2_ and were separated from the solution during filtration. No similar accumulations were observed in the corresponding dye solutions without adsorbent. Therefore, the measured decrease in dye concentration in the solution phase may include contributions from both surface uptake and adsorbent-associated aggregation phenomena. Since dye removal was based on the decrease in solution concentration after filtration, adsorption cannot be clearly separated from adsorbent induced aggregation or flocculation. Therefore, the calculated adsorption values can be regarded as apparent removal capacities under the applied experimental conditions.

## Conclusion

The synthesized Ba_2_Mg(BO_3_)_2_ was characterized using various analytical techniques and then evaluated for the removal of the cationic dyes MG and CRV from aqueous solutions under systematically varied experimental conditions. The effects of pH (4.5–10.5), adsorbent dosage (0.005–0.02 g per 30 mL), contact time (60–240 min), temperature (25–55 °C) and initial dye concentration (25–750 mg L^−1^) were investigated in detail. For both dyes, optimal adsorption performance was obtained under slightly alkaline and alkaline pH conditions. The use of 0.01 g of adsorbent showed that high removal efficiencies could be achieved even at relatively low dosage levels.

Before the main experiments, preliminary trials were conducted using higher Ba_2_Mg(BO_3_)_2_ dosages (0.025, 0.05, and 0.1 g) and various concentration ranges. High removal efficiencies were consistently observed even at elevated concentrations and dosages. Therefore, the main study focused on lower adsorbent amounts (0.005, 0.01, 0.015, and 0.02 g per 30 mL) and a defined concentration range of 25–750 mg L^−1^. Contact time studies showed that MG and CRV adsorption proceeded rapidly during the initial stage and reached equilibrium at approximately 120 min. Adsorption efficiency increased slightly but consistently with temperature, supporting the endothermic nature of the process. Particle size analysis showed a broad distribution (SPAN = 2.02) and a polydisperse character. The wide *D*10–*D*90 range indicated the presence of both fine and medium-sized particle fractions in the Ba_2_Mg(BO_3_)_2_ sample.

The PSO model provided the best empirical fit for both MG and CRV. Nonlinear Langmuir analysis yielded *q*_max_ values of 2283.835 and 2327.393 mg g^−1^ for MG and CRV, respectively. High adsorption capacities were observed despite the relatively low BET surface area of 1.3244 m^2^ g^−1^. The surface area determined by N_2_ adsorption at 77 K reflects physical adsorption under dry conditions, whereas dye adsorption occurs in an aqueous medium under substantially different physicochemical conditions. The good agreement with the nonlinear Langmuir model indicates Langmuir-type equilibrium behavior within the investigated concentration range. Therefore, the adsorption performance cannot be interpreted solely on the basis of the BET surface area. The successful synthesis process, high adsorption capacity, and strong removal performance indicate that Ba_2_Mg(BO_3_)_2_ has considerable potential for MG and CRV removal. Although the present study was conducted under laboratory conditions, the consistent results suggest that further investigation at larger scales may be warranted.

## Consent for publication

All authors approved the final version of the manuscript.

## Author contributions

U. Özkan: conceptualization, formal analysis, resources, methodology, investigation, writing – original draft, writing – review and editing, software, validation, visualization. O. Bayram: conceptualization, investigation, methodology, supervision. F. Göde: data curation, investigation, conceptualization, formal analysis, project administration, writing – original draft, writing – review and editing. İ. Pekgözlü: conceptualization, data curation, investigation, validation, visualization, writing – original draft, writing – review and editing.

## Conflicts of interest

The authors declare that they have no conflicts of interest.

## Data Availability

Data will be available on reasonable request.
